# Hydro­nium bis­(tri­fluoro­methane­sulfon­yl)amide–18-crown-6 (1/1)

**DOI:** 10.1107/S2414314620001625

**Published:** 2020-02-06

**Authors:** Atsushi Kitada, Yusuke Funasako, Kazuhiko Matsumoto, Rika Hagiwara, Makoto Inokuchi, Kazuhiro Fukami, Kuniaki Murase

**Affiliations:** a Department of Materials Science and Engineering, Kyoto University, 36-1, Yoshida-honmachi, Sakyo-ku, Kyoto 606-8501, Japan; b Department of Applied Chemistry, Faculty of Engineering, Sanyo-Onoda City University, 1-1-1, Daigakudori, Sanyo-Onoda, Yamaguchi, 756-0884, Japan; c Department of Fundamental Energy Science, Graduate School of Energy Science, Kyoto University, 36-1, Yoshida-honmachi, Sakyo-ku, Kyoto 606-8501, Japan; Okayama University, Japan

**Keywords:** crystal structure, hydro­nium, crown ether, complex cation, ionic liquid

## Abstract

The structure of the title compound, H_3_O^+^·C_2_F_6_NO_4_S_2_^−^·C_12_H_24_O_6_ or [H_3_O^+^·C_12_H_24_O_6_][N(SO_2_CF_3_)_2_^−^], which is an ionic liquid with a melting point of 341–343 K, has been determined at 113 K. The asymmetric unit consists of two crystallographically independent 18-crown-6 mol­ecules, two hydro­nium ions and two bis­(tri­fluoro­methane­sulfon­yl)amide anions; each 18-crown-6 mol­ecule complexes with a hydro­nium ion. In one 18-crown-6 mol­ecule, a part of the ring exhibits conformational disorder over two sets of sites with an occupancy ratio of 0.533 (13):0.467 (13). One hydro­nium ion is complexed with the ordered 18-crown-6 mol­ecule *via* O—H⋯O hydrogen bonds with H_2_OH⋯OC distances of 1.90 (6)–2.19 (7) Å, and the other hydro­nium ion with the disordered crown mol­ecule with distances of 1.85 (6)–2.36 (6) Å. The hydro­nium ions are also linked to the anions *via* O—H⋯F hydrogen bonds. The crystal studied was found to be a racemic twin with a component ratio of 0.55 (13):0.45 (13).

## Abstract 2

The structure of the title compound, [H_3_O^+^·C_12_H_24_O_6_][N(SO_2_CF_3_)_2_^−^], known as an ionic liquid (m.p. 341 −343 K), has been determined at 113 K. One hydro­nium ion is complexed with an ordered 18-crown-6 mol­ecule with H_2_OH⋯OC distances of 1.90–2.19 Å, and another hydro­nium ion with a disordered 18-crown-6 mol­ecule with distances of 1.85–2.36 Å.

## Graphical abstract

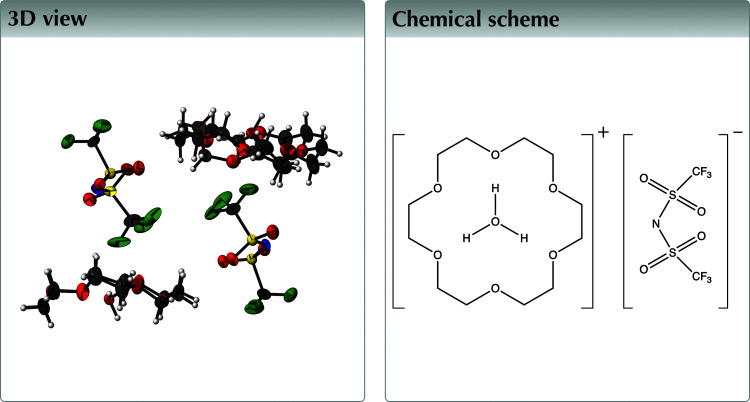

## Structure description

**Figure 1:**
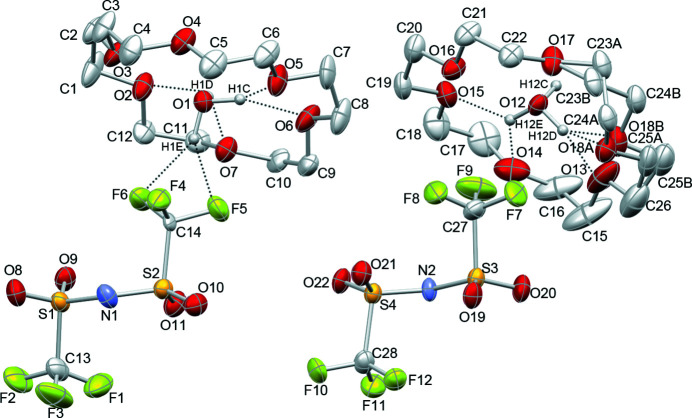
The asymmetric unit of the title compound, showing the two crystallographically independent ion-pairs. Displacement ellipsoids are shown at the 50% probability level and hydrogen atoms of 18-crown-6 are omitted for clarity. Dashed lines represent hydrogen bonds.

**Figure 2:**
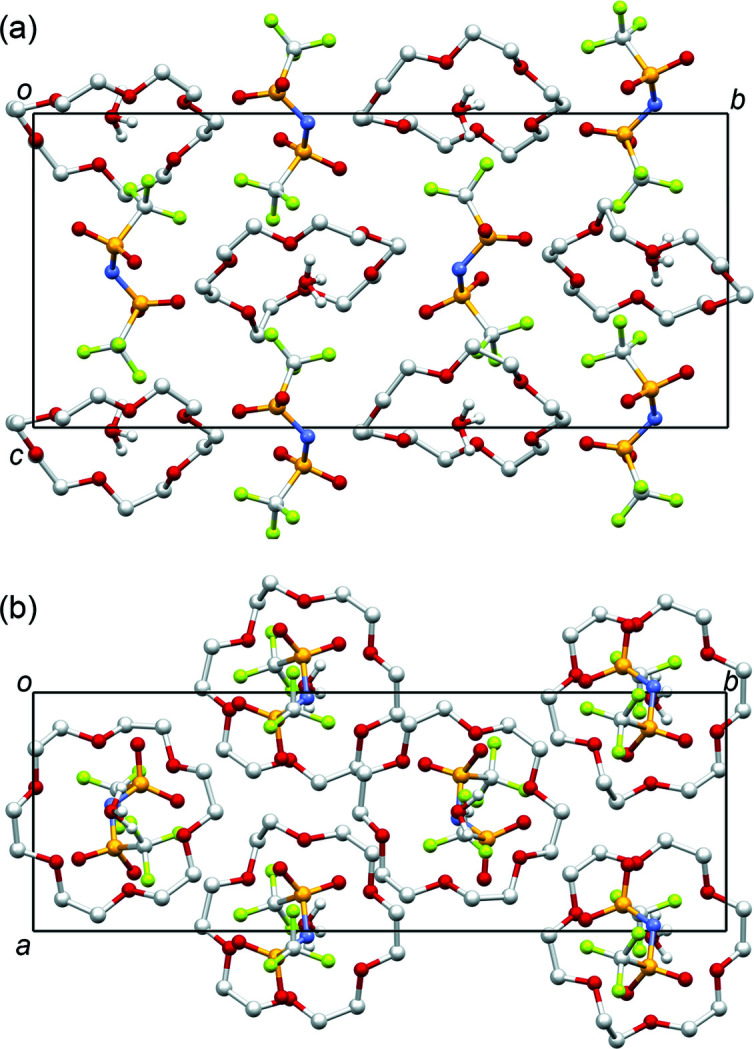
Structure of the title compound viewed along (*a*) the *a* axis and (*b*) the *c* axis. Hydrogen atoms of 18-crown-6 are omitted for clarity.

Hydro­nium·18-crown-6 bis­(tri­fluoro­methane­sulfon­yl)amide is an ionized form of ternary equimolar mixture of 18-crown-6, imide superacid and water, the molten salt of which is known as a hydro­nium solvate ionic liquid (m.p. 341 −343 K) with very strong Brønsted acidity (Kitada *et al.*, 2018 ▸). The title compound crystallizes in the monoclinic space group *P*2_1_. The asymmetric unit contains two crystallographically independent ion-pairs (Fig. 1 ▸); each 18-crown-6 mol­ecule complexes with a hydro­nium ion. One of the 18-crown-6 mol­ecules exhibits conformational disorder. The two bis­(tri­fluoro­methane­sulfon­yl)amide anions adopt a *transoid* conformation. The complex cations and anions are arranged alternately along the *c* axis to form columns (Fig. 2 ▸). One hydro­nium ion is complexed with the ordered 18-crown-6 mol­ecule *via* O—H⋯O hydrogen bonds with H_2_OH⋯OC distances of 1.90 (6)–2.19 (7) Å, and the other hydro­nium ion with the disordered 18-crown-6 mol­ecule with 1.85 (6)–2.36 (6) Å distances (Table 1 ▸). The hydro­nium ion complexed with the ordered crown inter­acts with two anions *via* O—H⋯F hydrogen bonds with H_2_OH⋯F_3_C distances of 2.12 (4)–2.14 (6) Å, while the hydro­nium ion with the disordered crown exhibits a weak O—H⋯F inter­action [H⋯F = 2.50 (4) Å].

## Synthesis and crystallization

The polycrystalline title compound was synthesized according to a previous report (Kitada *et al.*, 2018 ▸). The powder sample was dissolved in copious amounts of water and stored in a plastic-wrapped Petri dish. Single crystals of the title compound were prepared by slow evaporation as colorless prisms.

## Refinement

Crystal data, data collection and structure refinement details are summarized in Table 2 ▸. The structure was refined as an inversion twin. Restraints (*SADI*, *DFIX*, *DANG*, *SIMU* and *ISOR*) were used to correct the geometry of the disordered crown ether mol­ecule and hydro­nium ion, and the displacement parameters of the disordered crown ether mol­ecule.

## Supplementary Material

Crystal structure: contains datablock(s) I. DOI: 10.1107/S2414314620001625/is4042sup1.cif

Structure factors: contains datablock(s) I. DOI: 10.1107/S2414314620001625/is4042Isup2.hkl

CCDC reference: 1982024

Additional supporting information:  crystallographic information; 3D view; checkCIF report

## Figures and Tables

**Table 1:** Hydrogen-bond geometry (Å, °)

*D*—H⋯*A*	*D*—H	H⋯*A*	*D*⋯*A*	*D*—H⋯*A*
O1—H1*C*⋯O5	1.00 (3)	1.90 (6)	2.690 (7)	134 (6)
O1—H1*C*⋯O6	1.00 (3)	2.08 (6)	2.898 (8)	138 (6)
O1—H1*D*⋯O2	1.01 (3)	2.19 (7)	2.835 (7)	120 (5)
O1—H1*D*⋯O7	1.01 (3)	1.96 (7)	2.687 (7)	127 (6)
O1—H1*E*⋯F5^i^	1.03 (3)	2.14 (6)	2.989 (7)	139 (6)
O1—H1*E*⋯F6^i^	1.03 (3)	2.12 (4)	3.065 (7)	152 (6)
O12—H12*C*⋯F11^ii^	1.00 (3)	2.50 (4)	3.436 (8)	154 (7)
O12—H12*D*⋯O13	0.97 (3)	1.85 (6)	2.666 (9)	140 (7)
O12—H12*D*⋯O18*A*	0.97 (3)	2.36 (6)	3.079 (12)	131 (6)
O12—H12*D*⋯O18*B*	0.97 (3)	1.97 (6)	2.753 (15)	137 (7)
O12—H12*E*⋯O14	0.97 (3)	2.14 (7)	2.831 (8)	128 (6)
O12—H12*E*⋯O15	0.97 (3)	1.95 (6)	2.718 (7)	135 (6)

Symmetry codes: (i) 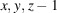 ; (ii) 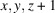 .

**Table 2:** Experimental details

Crystal data	
Chemical formula	H_3_O^+^·C_2_F_6_NO_4_S_2_^−^·C_12_H_24_O_6_
*M*_r_	563.48
Crystal system, space group	Monoclinic, *P*2_1_
Temperature (K)	113
*a*, *b*, *c* (Å)	8.8341 (4), 24.3932 (9), 11.6111 (5)
β (°)	108.086 (2)
*V* (Å^3^)	2378.47 (17)
*Z*	4
Radiation type	Mo *K*α
μ (mm^−1^)	0.32
Crystal size (mm)	0.50 × 0.30 × 0.20

Data collection	
Diffractometer	Rigaku R-AXIS RAPID-II
Absorption correction	Multi-scan (*ABSCOR*; Higashi, 1995 ▸)
*T*_min_, *T*_max_	0.768, 1.000
No. of measured, independent and observed [*I* > 2σ(*I*)] reflections	20448, 9354, 8989
*R*_int_	0.027
(sin θ/λ)_max_ (Å^−1^)	0.617

Refinement	
*R*[*F* ^2^ > 2σ(*F* ^2^)], *wR*(*F* ^2^), *S*	0.062, 0.162, 1.10
No. of reflections	9354
No. of parameters	669
No. of restraints	217
H-atom treatment	H atoms treated by a mixture of independent and constrained refinement
Δρ_max_, Δρ_min_ (e Å^−3^)	1.00, −0.54
Absolute structure	Refined as an inversion twin
Absolute structure parameter	0.45 (13)

Computer programs: *RAPID-AUTO* (Rigaku, 2006 ▸), *SHELXT* (Sheldrick, 2015*a* ▸) and *SHELXL2018* (Sheldrick, 2015*b* ▸).

## full crystallographic data

### Crystal data

**Table:**

H_3_O^+^·C_2_F_6_NO_4_S_2_^−^·C_12_H_24_O_6_	*F*(000) = 1168
*M**_r_* = 563.48	*D*_x_ = 1.574 Mg m^−^^3^
Monoclinic, *P*2_1_	Mo *K*α radiation, λ = 0.71073 Å
*a* = 8.8341 (4) Å	Cell parameters from 22988 reflections
*b* = 24.3932 (9) Å	θ = 3.1–27.6°
*c* = 11.6111 (5) Å	µ = 0.32 mm^−^^1^
β = 108.086 (2)°	*T* = 113 K
*V* = 2378.47 (17) Å^3^	Block, colorless
*Z* = 4	0.50 × 0.30 × 0.20 mm

### Data collection

**Table:**

Rigaku R-AXIS RAPID-II diffractometer	8989 reflections with *I* > 2σ(*I*)
Radiation source: fine-focus sealed tube	*R*_int_ = 0.027
imaging plate scans	θ_max_ = 26.0°, θ_min_ = 3.1°
Absorption correction: multi-scan (*ABSCOR*; Higashi, 1995)	*h* = −10→10
*T*_min_ = 0.768, *T*_max_ = 1.000	*k* = −30→30
20448 measured reflections	*l* = −14→14
9354 independent reflections	

### Refinement

**Table:**

Refinement on *F*^2^	Hydrogen site location: mixed
Least-squares matrix: full	H atoms treated by a mixture of independent and constrained refinement
*R*[*F*^2^ > 2σ(*F*^2^)] = 0.062	*w* = 1/[σ^2^(*F*_o_^2^) + (0.065*P*)^2^ + 5.3126*P*] where *P* = (*F*_o_^2^ + 2*F*_c_^2^)/3
*wR*(*F*^2^) = 0.162	(Δ/σ)_max_ < 0.001
*S* = 1.10	Δρ_max_ = 1.00 e Å^−^^3^
9354 reflections	Δρ_min_ = −0.53 e Å^−^^3^
669 parameters	Absolute structure: Refined as an inversion twin
217 restraints	Absolute structure parameter: 0.45 (13)

### Special details

**Table:**

Geometry. All esds (except the esd in the dihedral angle between two l.s. planes) are estimated using the full covariance matrix. The cell esds are taken into account individually in the estimation of esds in distances, angles and torsion angles; correlations between esds in cell parameters are only used when they are defined by crystal symmetry. An approximate (isotropic) treatment of cell esds is used for estimating esds involving l.s. planes.
Refinement. Refined as a 2-component inversion twin.

### Fractional atomic coordinates and isotropic or equivalent isotropic displacement parameters (Å^2^)

**Table:**

	*x*	*y*	*z*	*U*_iso_*/*U*_eq_	Occ. (<1)
C1	0.0670 (10)	0.0933 (4)	−0.1013 (12)	0.066 (3)	
H1A	0.057097	0.087881	−0.187835	0.079*	
H1AB	−0.040231	0.101149	−0.095175	0.079*	
C2	0.1339 (9)	0.0420 (4)	−0.0300 (9)	0.047 (2)	
H2A	0.141282	0.046852	0.056193	0.057*	
H2AB	0.064038	0.010248	−0.062655	0.057*	
C3	0.3396 (10)	−0.0224 (3)	−0.0127 (10)	0.050 (2)	
H3A	0.268997	−0.047567	−0.072547	0.060*	
H3AB	0.333858	−0.032033	0.068611	0.060*	
C4	0.5062 (11)	−0.0282 (3)	−0.0151 (10)	0.052 (2)	
H4A	0.539579	−0.067114	−0.005627	0.063*	
H4AB	0.516047	−0.014386	−0.092686	0.063*	
C5	0.7642 (10)	0.0035 (4)	0.0872 (9)	0.050 (2)	
H5A	0.774811	0.017887	0.010397	0.059*	
H5AB	0.807994	−0.034182	0.099784	0.059*	
C6	0.8502 (9)	0.0395 (3)	0.1906 (9)	0.049 (2)	
H6A	0.829959	0.026958	0.265622	0.058*	
H6AB	0.966225	0.037710	0.203504	0.058*	
C7	0.8638 (10)	0.1330 (4)	0.2572 (9)	0.056 (2)	
H7A	0.976004	0.123169	0.299674	0.067*	
H7AB	0.804551	0.133303	0.316916	0.067*	
C8	0.8551 (10)	0.1875 (4)	0.1995 (11)	0.060 (3)	
H8A	0.909389	0.215113	0.260847	0.072*	
H8AB	0.909252	0.186324	0.136546	0.072*	
C9	0.6681 (10)	0.2461 (3)	0.0631 (8)	0.0428 (19)	
H9A	0.697188	0.234582	−0.009044	0.051*	
H9AB	0.736510	0.277427	0.101176	0.051*	
C10	0.4992 (10)	0.2626 (3)	0.0267 (8)	0.0407 (18)	
H10A	0.469132	0.272758	0.099187	0.049*	
H10B	0.482402	0.294913	−0.027434	0.049*	
C11	0.2374 (9)	0.2310 (3)	−0.0634 (8)	0.0420 (18)	
H11A	0.214336	0.264912	−0.112523	0.050*	
H11B	0.211561	0.237637	0.012539	0.050*	
C12	0.1387 (9)	0.1853 (3)	−0.1316 (8)	0.0408 (18)	
H12A	0.024278	0.194943	−0.154358	0.049*	
H12B	0.166127	0.177644	−0.206403	0.049*	
C13	0.3570 (10)	0.1603 (5)	0.2955 (8)	0.052 (2)	
C14	0.5924 (8)	0.1220 (3)	0.7379 (6)	0.0290 (14)	
O1	0.5005 (6)	0.1132 (2)	0.0107 (5)	0.0304 (10)	
H1C	0.598 (6)	0.127 (3)	0.073 (5)	0.046*	
H1D	0.431 (7)	0.142 (3)	0.028 (6)	0.046*	
H1E	0.500 (9)	0.128 (3)	−0.072 (4)	0.046*	
O2	0.1713 (6)	0.1382 (2)	−0.0534 (6)	0.0453 (14)	
O3	0.2887 (6)	0.0330 (2)	−0.0412 (6)	0.0431 (13)	
O4	0.6016 (7)	0.0036 (2)	0.0837 (6)	0.0436 (13)	
O5	0.7946 (6)	0.0945 (2)	0.1632 (6)	0.0490 (15)	
O6	0.6923 (6)	0.2022 (2)	0.1460 (6)	0.0497 (15)	
O7	0.4007 (6)	0.2173 (2)	−0.0360 (5)	0.0367 (12)	
O8	0.3018 (7)	0.0657 (2)	0.3675 (5)	0.0420 (13)	
O9	0.2436 (6)	0.1455 (2)	0.4735 (5)	0.0382 (12)	
O10	0.7819 (6)	0.1506 (2)	0.6270 (5)	0.0366 (11)	
O11	0.5448 (6)	0.2078 (2)	0.6002 (5)	0.0420 (13)	
N1	0.5279 (7)	0.1133 (3)	0.5041 (6)	0.0365 (14)	
F1	0.4093 (7)	0.2104 (3)	0.3319 (6)	0.078 (2)	
F2	0.2132 (7)	0.1654 (3)	0.2160 (5)	0.0706 (18)	
F3	0.4538 (8)	0.1396 (4)	0.2414 (5)	0.086 (2)	
F4	0.6419 (6)	0.0728 (2)	0.7592 (4)	0.0472 (12)	
F5	0.6679 (6)	0.1527 (3)	0.8387 (4)	0.0525 (12)	
F6	0.4344 (5)	0.1229 (3)	0.7363 (4)	0.0526 (13)	
S1	0.3486 (2)	0.11734 (8)	0.42120 (16)	0.0311 (4)	
S2	0.61456 (19)	0.15466 (7)	0.60634 (16)	0.0296 (4)	
C15	0.0757 (19)	0.5241 (4)	0.4202 (18)	0.109 (6)	
H15A	0.077681	0.512169	0.339121	0.131*	
H15B	0.122820	0.561191	0.436108	0.131*	
C16	−0.0919 (18)	0.5251 (5)	0.4239 (15)	0.095 (5)	
H16A	−0.093031	0.531839	0.507709	0.114*	
H16B	−0.152353	0.554761	0.371332	0.114*	
C17	−0.3160 (12)	0.4672 (4)	0.3958 (11)	0.067 (3)	
H17A	−0.388080	0.496340	0.350597	0.080*	
H17B	−0.309048	0.469928	0.482380	0.080*	
C18	−0.3782 (12)	0.4123 (5)	0.3473 (10)	0.064 (3)	
H18A	−0.488847	0.408243	0.349433	0.077*	
H18B	−0.380018	0.409422	0.261863	0.077*	
C19	−0.3272 (9)	0.3177 (3)	0.3598 (8)	0.0405 (18)	
H19A	−0.300690	0.315790	0.283080	0.049*	
H19B	−0.443201	0.311850	0.341198	0.049*	
C20	−0.2390 (10)	0.2752 (4)	0.4442 (9)	0.0459 (19)	
H20A	−0.265237	0.277281	0.521009	0.055*	
H20B	−0.270004	0.238405	0.408478	0.055*	
C21	0.0189 (9)	0.2441 (3)	0.5509 (9)	0.0432 (19)	
H21A	−0.002599	0.206788	0.515921	0.052*	
H21B	−0.010505	0.244962	0.626550	0.052*	
C22	0.1917 (10)	0.2577 (3)	0.5778 (8)	0.0415 (18)	
H22A	0.258138	0.229553	0.631856	0.050*	
H22B	0.220123	0.258542	0.501743	0.050*	
C23A	0.3782 (13)	0.3235 (7)	0.7054 (12)	0.042 (4)	0.533 (13)
H23A	0.433543	0.291189	0.750923	0.051*	0.533 (13)
H23B	0.377976	0.353132	0.763601	0.051*	0.533 (13)
C23B	0.3882 (13)	0.3211 (6)	0.660 (2)	0.042 (4)	0.467 (13)
H23C	0.419774	0.313505	0.587191	0.050*	0.467 (13)
H23D	0.450638	0.296619	0.725983	0.050*	0.467 (13)
C25A	0.411 (2)	0.4465 (6)	0.6067 (16)	0.057 (3)	0.533 (13)
H25A	0.526860	0.454000	0.630793	0.068*	0.533 (13)
H25B	0.377277	0.449189	0.680189	0.068*	0.533 (13)
C25B	0.414 (3)	0.4658 (8)	0.6330 (16)	0.066 (4)	0.467 (13)
H25C	0.530931	0.467396	0.649552	0.079*	0.467 (13)
H25D	0.383820	0.480591	0.702106	0.079*	0.467 (13)
C26	0.3242 (14)	0.4894 (5)	0.5167 (12)	0.081 (3)	
H26A	0.367593	0.526319	0.543631	0.098*	0.533 (13)
H26B	0.336231	0.482085	0.436116	0.098*	0.533 (13)
H26C	0.356390	0.527986	0.511347	0.098*	0.467 (13)
H26D	0.342946	0.468455	0.449385	0.098*	0.467 (13)
C27	0.1148 (9)	0.3490 (4)	0.2430 (8)	0.0421 (18)	
C28	−0.0718 (9)	0.3754 (3)	−0.2055 (7)	0.0379 (17)	
O12	0.0255 (6)	0.3927 (2)	0.5372 (5)	0.0325 (11)	
H12C	−0.003 (9)	0.414 (3)	0.601 (5)	0.049*	
H12D	0.113 (6)	0.414 (3)	0.529 (7)	0.049*	
H12E	−0.066 (6)	0.398 (3)	0.467 (4)	0.049*	
O13	0.1651 (10)	0.4866 (3)	0.5106 (9)	0.078 (2)	
O14	−0.1628 (9)	0.4733 (3)	0.3824 (6)	0.0589 (18)	
O15	−0.2840 (7)	0.3701 (2)	0.4150 (5)	0.0429 (13)	
O16	−0.0720 (6)	0.2832 (2)	0.4676 (5)	0.0405 (12)	
O17	0.2186 (6)	0.3097 (2)	0.6346 (6)	0.0466 (14)	
O19	0.2612 (5)	0.3562 (2)	0.0831 (5)	0.0351 (11)	
O20	0.2028 (7)	0.4408 (2)	0.1757 (5)	0.0431 (13)	
O21	−0.0471 (6)	0.2968 (2)	−0.0511 (5)	0.0348 (11)	
O22	−0.2774 (5)	0.3584 (2)	−0.0922 (5)	0.0353 (11)	
C24A	0.4575 (15)	0.3420 (5)	0.6170 (12)	0.039 (3)	0.533 (13)
H24A	0.571588	0.349212	0.659214	0.047*	0.533 (13)
H24B	0.449811	0.313106	0.555715	0.047*	0.533 (13)
O18A	0.3818 (12)	0.3912 (4)	0.5588 (10)	0.042 (2)	0.533 (13)
C24B	0.4246 (19)	0.3799 (6)	0.6978 (13)	0.045 (3)	0.467 (13)
H24C	0.383639	0.389097	0.765684	0.053*	0.467 (13)
H24D	0.540929	0.386327	0.724032	0.053*	0.467 (13)
O18B	0.3470 (17)	0.4126 (6)	0.5934 (12)	0.050 (3)	0.467 (13)
N2	−0.0224 (6)	0.3950 (2)	0.0293 (5)	0.0267 (12)	
F7	0.2511 (7)	0.3449 (3)	0.3368 (5)	0.075 (2)	
F8	0.0674 (8)	0.2991 (2)	0.2124 (5)	0.0673 (17)	
F9	0.0113 (9)	0.3734 (3)	0.2863 (6)	0.083 (2)	
F10	−0.1444 (7)	0.3425 (3)	−0.2958 (4)	0.0616 (15)	
F11	−0.1256 (6)	0.4256 (2)	−0.2323 (5)	0.0513 (12)	
F12	0.0831 (5)	0.3750 (2)	−0.1900 (4)	0.0426 (11)	
S3	0.15173 (19)	0.38867 (7)	0.12153 (15)	0.0277 (4)	
S4	−0.10989 (18)	0.35095 (7)	−0.06740 (15)	0.0268 (3)	

### Atomic displacement parameters (Å^2^)

**Table:**

	*U*^11^	*U*^22^	*U*^33^	*U*^12^	*U*^13^	*U*^23^
C1	0.023 (4)	0.047 (5)	0.107 (9)	−0.009 (4)	−0.009 (5)	0.011 (5)
C2	0.019 (3)	0.044 (5)	0.076 (6)	−0.005 (3)	0.011 (4)	0.007 (4)
C3	0.039 (4)	0.021 (4)	0.091 (7)	−0.006 (3)	0.020 (4)	−0.007 (4)
C4	0.051 (5)	0.031 (4)	0.074 (6)	−0.004 (4)	0.020 (5)	−0.013 (4)
C5	0.036 (4)	0.037 (4)	0.076 (6)	0.011 (3)	0.018 (4)	0.005 (4)
C6	0.026 (4)	0.039 (4)	0.077 (6)	0.006 (3)	0.009 (4)	0.014 (4)
C7	0.029 (4)	0.058 (5)	0.064 (6)	−0.010 (4)	−0.011 (4)	−0.015 (4)
C8	0.026 (4)	0.044 (5)	0.094 (8)	−0.006 (4)	−0.003 (4)	−0.008 (5)
C9	0.049 (5)	0.035 (4)	0.046 (5)	−0.015 (3)	0.019 (4)	−0.005 (3)
C10	0.052 (5)	0.021 (4)	0.054 (5)	−0.007 (3)	0.023 (4)	−0.008 (3)
C11	0.033 (4)	0.042 (4)	0.048 (5)	0.010 (3)	0.008 (3)	0.012 (3)
C12	0.030 (4)	0.041 (4)	0.044 (4)	0.006 (3)	−0.001 (3)	0.010 (3)
C13	0.032 (4)	0.083 (7)	0.033 (4)	−0.007 (4)	0.001 (3)	0.008 (4)
C14	0.022 (3)	0.037 (4)	0.019 (3)	−0.010 (3)	−0.007 (2)	0.005 (3)
O1	0.027 (2)	0.030 (3)	0.032 (3)	0.003 (2)	0.0065 (19)	−0.002 (2)
O2	0.028 (3)	0.034 (3)	0.062 (4)	−0.002 (2)	−0.003 (2)	0.006 (3)
O3	0.032 (3)	0.031 (3)	0.069 (4)	−0.005 (2)	0.019 (3)	0.003 (3)
O4	0.033 (3)	0.034 (3)	0.067 (4)	0.003 (2)	0.020 (3)	−0.001 (3)
O5	0.027 (3)	0.040 (3)	0.066 (4)	−0.001 (2)	−0.005 (3)	−0.003 (3)
O6	0.027 (3)	0.041 (3)	0.078 (4)	−0.007 (2)	0.011 (3)	0.010 (3)
O7	0.024 (2)	0.034 (3)	0.052 (3)	0.004 (2)	0.011 (2)	−0.001 (2)
O8	0.034 (3)	0.047 (3)	0.037 (3)	−0.001 (2)	0.001 (2)	−0.009 (2)
O9	0.026 (2)	0.046 (3)	0.043 (3)	−0.003 (2)	0.012 (2)	−0.006 (2)
O10	0.024 (2)	0.042 (3)	0.043 (3)	0.005 (2)	0.009 (2)	0.007 (2)
O11	0.030 (3)	0.038 (3)	0.055 (4)	0.001 (2)	0.009 (2)	0.002 (3)
N1	0.029 (3)	0.050 (4)	0.028 (3)	0.005 (3)	0.005 (3)	−0.006 (3)
F1	0.063 (4)	0.086 (5)	0.071 (4)	−0.026 (3)	0.002 (3)	0.038 (4)
F2	0.044 (3)	0.115 (5)	0.040 (3)	0.002 (3)	−0.005 (2)	0.025 (3)
F3	0.060 (4)	0.163 (8)	0.045 (3)	0.011 (4)	0.032 (3)	0.017 (4)
F4	0.042 (3)	0.049 (3)	0.044 (3)	−0.005 (2)	0.003 (2)	0.014 (2)
F5	0.045 (3)	0.078 (4)	0.030 (2)	−0.010 (3)	0.006 (2)	−0.009 (2)
F6	0.029 (2)	0.092 (4)	0.037 (3)	−0.012 (2)	0.0116 (19)	0.001 (2)
S1	0.0234 (8)	0.0427 (10)	0.0258 (9)	−0.0008 (7)	0.0053 (6)	0.0001 (7)
S2	0.0212 (8)	0.0355 (9)	0.0309 (9)	0.0007 (7)	0.0065 (6)	0.0022 (7)
C15	0.145 (13)	0.029 (5)	0.212 (19)	0.002 (6)	0.139 (14)	0.010 (8)
C16	0.136 (12)	0.044 (6)	0.150 (13)	0.023 (7)	0.110 (11)	0.032 (7)
C17	0.047 (5)	0.064 (7)	0.085 (8)	0.033 (5)	0.014 (5)	0.004 (5)
C18	0.048 (5)	0.064 (6)	0.066 (6)	0.011 (5)	−0.002 (5)	0.000 (5)
C19	0.028 (4)	0.047 (5)	0.040 (4)	−0.004 (3)	0.002 (3)	−0.013 (3)
C20	0.035 (4)	0.045 (5)	0.056 (5)	−0.006 (3)	0.012 (4)	−0.011 (4)
C21	0.034 (4)	0.031 (4)	0.066 (6)	0.006 (3)	0.018 (4)	0.009 (4)
C22	0.037 (4)	0.035 (4)	0.052 (5)	0.006 (3)	0.012 (4)	0.006 (3)
C23A	0.029 (5)	0.051 (6)	0.038 (7)	0.005 (5)	−0.003 (5)	−0.010 (6)
C23B	0.031 (6)	0.044 (6)	0.038 (7)	0.008 (5)	−0.007 (6)	−0.005 (6)
C25A	0.040 (5)	0.070 (6)	0.064 (6)	−0.023 (5)	0.019 (5)	−0.030 (5)
C25B	0.052 (6)	0.076 (7)	0.076 (7)	−0.022 (6)	0.026 (6)	−0.024 (6)
C26	0.071 (5)	0.073 (6)	0.117 (6)	−0.030 (4)	0.054 (5)	−0.028 (5)
C27	0.034 (4)	0.052 (5)	0.042 (4)	−0.011 (4)	0.014 (3)	0.006 (4)
C28	0.031 (4)	0.046 (4)	0.040 (4)	−0.007 (3)	0.016 (3)	−0.010 (3)
O12	0.024 (2)	0.036 (3)	0.040 (3)	−0.006 (2)	0.013 (2)	−0.002 (2)
O13	0.075 (5)	0.064 (4)	0.120 (6)	−0.027 (4)	0.067 (4)	−0.022 (4)
O14	0.074 (5)	0.052 (4)	0.062 (4)	0.027 (3)	0.037 (4)	0.018 (3)
O15	0.037 (3)	0.048 (3)	0.038 (3)	0.001 (2)	0.003 (2)	−0.006 (2)
O16	0.028 (3)	0.041 (3)	0.051 (3)	0.001 (2)	0.010 (2)	0.010 (2)
O17	0.038 (3)	0.034 (3)	0.056 (4)	0.007 (2)	−0.001 (3)	−0.001 (2)
O19	0.018 (2)	0.039 (3)	0.046 (3)	0.002 (2)	0.006 (2)	−0.008 (2)
O20	0.040 (3)	0.033 (3)	0.049 (3)	−0.010 (2)	0.003 (3)	−0.010 (2)
O21	0.028 (3)	0.032 (3)	0.042 (3)	0.002 (2)	0.007 (2)	−0.004 (2)
O22	0.018 (2)	0.039 (3)	0.046 (3)	−0.004 (2)	0.005 (2)	−0.004 (2)
C24A	0.024 (5)	0.049 (5)	0.038 (5)	0.003 (4)	0.000 (4)	−0.008 (5)
O18A	0.030 (4)	0.046 (5)	0.048 (5)	−0.004 (4)	0.008 (4)	−0.002 (4)
C24B	0.031 (5)	0.053 (5)	0.045 (5)	−0.002 (5)	0.004 (4)	−0.011 (5)
O18B	0.039 (5)	0.056 (6)	0.050 (5)	−0.003 (5)	0.004 (4)	−0.015 (5)
N2	0.017 (3)	0.026 (3)	0.030 (3)	0.003 (2)	−0.002 (2)	−0.006 (2)
F7	0.064 (4)	0.093 (5)	0.041 (3)	−0.027 (3)	−0.021 (3)	0.026 (3)
F8	0.094 (4)	0.053 (3)	0.046 (3)	−0.035 (3)	0.009 (3)	0.008 (2)
F9	0.093 (5)	0.112 (6)	0.065 (4)	0.019 (4)	0.054 (4)	0.020 (4)
F10	0.072 (4)	0.080 (4)	0.030 (3)	−0.035 (3)	0.012 (2)	−0.019 (2)
F11	0.051 (3)	0.051 (3)	0.046 (3)	−0.003 (2)	0.006 (2)	0.014 (2)
F12	0.037 (2)	0.055 (3)	0.041 (3)	−0.009 (2)	0.0194 (19)	−0.005 (2)
S3	0.0204 (8)	0.0291 (8)	0.0316 (9)	−0.0015 (6)	0.0050 (6)	−0.0026 (7)
S4	0.0195 (7)	0.0288 (8)	0.0306 (8)	−0.0002 (6)	0.0056 (6)	−0.0017 (6)

### Geometric parameters (Å, º)

**Table:**

C1—O2	1.428 (10)	C17—O14	1.418 (12)
C1—C2	1.515 (13)	C17—C18	1.489 (15)
C1—H1A	0.9900	C17—H17A	0.9900
C1—H1AB	0.9900	C17—H17B	0.9900
C2—O3	1.430 (9)	C18—O15	1.401 (11)
C2—H2A	0.9900	C18—H18A	0.9900
C2—H2AB	0.9900	C18—H18B	0.9900
C3—O3	1.431 (9)	C19—O15	1.428 (9)
C3—C4	1.487 (12)	C19—C20	1.473 (12)
C3—H3A	0.9900	C19—H19A	0.9900
C3—H3AB	0.9900	C19—H19B	0.9900
C4—O4	1.423 (11)	C20—O16	1.428 (9)
C4—H4A	0.9900	C20—H20A	0.9900
C4—H4AB	0.9900	C20—H20B	0.9900
C5—O4	1.425 (10)	C21—O16	1.418 (9)
C5—C6	1.490 (13)	C21—C22	1.497 (11)
C5—H5A	0.9900	C21—H21A	0.9900
C5—H5AB	0.9900	C21—H21B	0.9900
C6—O5	1.432 (10)	C22—O17	1.416 (10)
C6—H6A	0.9900	C22—H22A	0.9900
C6—H6AB	0.9900	C22—H22B	0.9900
C7—O5	1.426 (10)	C23A—O17	1.435 (11)
C7—C8	1.479 (14)	C23A—C24A	1.480 (11)
C7—H7A	0.9900	C23A—H23A	0.9900
C7—H7AB	0.9900	C23A—H23B	0.9900
C8—O6	1.424 (10)	C23B—O17	1.461 (11)
C8—H8A	0.9900	C23B—C24B	1.504 (13)
C8—H8AB	0.9900	C23B—H23C	0.9900
C9—O6	1.410 (10)	C23B—H23D	0.9900
C9—C10	1.475 (12)	C25A—O18A	1.451 (12)
C9—H9A	0.9900	C25A—C26	1.510 (14)
C9—H9AB	0.9900	C25A—H25A	0.9900
C10—O7	1.454 (9)	C25A—H25B	0.9900
C10—H10A	0.9900	C25B—O18B	1.440 (12)
C10—H10B	0.9900	C25B—C26	1.457 (14)
C11—O7	1.418 (9)	C25B—H25C	0.9900
C11—C12	1.483 (12)	C25B—H25D	0.9900
C11—H11A	0.9900	C26—O13	1.387 (13)
C11—H11B	0.9900	C26—H26A	0.9900
C12—O2	1.438 (9)	C26—H26B	0.9900
C12—H12A	0.9900	C26—H26C	0.9900
C12—H12B	0.9900	C26—H26D	0.9900
C13—F3	1.309 (11)	C27—F8	1.300 (10)
C13—F2	1.324 (9)	C27—F9	1.315 (11)
C13—F1	1.329 (12)	C27—F7	1.353 (9)
C13—S1	1.816 (9)	C27—S3	1.822 (8)
C14—F4	1.277 (9)	C28—F11	1.316 (10)
C14—F5	1.374 (8)	C28—F10	1.318 (9)
C14—F6	1.390 (8)	C28—F12	1.324 (9)
C14—S2	1.786 (7)	C28—S4	1.837 (8)
O1—H1C	1.00 (3)	O12—H12C	1.00 (3)
O1—H1D	1.01 (3)	O12—H12D	0.97 (3)
O1—H1E	1.03 (3)	O12—H12E	0.97 (3)
O8—S1	1.408 (6)	O19—S3	1.424 (5)
O9—S1	1.432 (5)	O20—S3	1.429 (5)
O10—S2	1.426 (5)	O21—S4	1.422 (5)
O11—S2	1.427 (6)	O22—S4	1.429 (5)
N1—S2	1.566 (7)	C24A—O18A	1.436 (11)
N1—S1	1.580 (6)	C24A—H24A	0.9900
C15—O13	1.430 (18)	C24A—H24B	0.9900
C15—C16	1.495 (18)	C24B—O18B	1.436 (12)
C15—H15A	0.9900	C24B—H24C	0.9900
C15—H15B	0.9900	C24B—H24D	0.9900
C16—O14	1.425 (15)	N2—S4	1.572 (5)
C16—H16A	0.9900	N2—S3	1.586 (5)
C16—H16B	0.9900		
			
O2—C1—C2	108.9 (7)	O14—C17—H17A	110.1
O2—C1—H1A	109.9	C18—C17—H17A	110.1
C2—C1—H1A	109.9	O14—C17—H17B	110.1
O2—C1—H1AB	109.9	C18—C17—H17B	110.1
C2—C1—H1AB	109.9	H17A—C17—H17B	108.5
H1A—C1—H1AB	108.3	O15—C18—C17	111.3 (8)
O3—C2—C1	107.4 (7)	O15—C18—H18A	109.4
O3—C2—H2A	110.2	C17—C18—H18A	109.4
C1—C2—H2A	110.2	O15—C18—H18B	109.4
O3—C2—H2AB	110.2	C17—C18—H18B	109.4
C1—C2—H2AB	110.2	H18A—C18—H18B	108.0
H2A—C2—H2AB	108.5	O15—C19—C20	108.7 (6)
O3—C3—C4	109.1 (7)	O15—C19—H19A	110.0
O3—C3—H3A	109.9	C20—C19—H19A	110.0
C4—C3—H3A	109.9	O15—C19—H19B	110.0
O3—C3—H3AB	109.9	C20—C19—H19B	110.0
C4—C3—H3AB	109.9	H19A—C19—H19B	108.3
H3A—C3—H3AB	108.3	O16—C20—C19	109.4 (7)
O4—C4—C3	105.9 (7)	O16—C20—H20A	109.8
O4—C4—H4A	110.6	C19—C20—H20A	109.8
C3—C4—H4A	110.6	O16—C20—H20B	109.8
O4—C4—H4AB	110.6	C19—C20—H20B	109.8
C3—C4—H4AB	110.6	H20A—C20—H20B	108.3
H4A—C4—H4AB	108.7	O16—C21—C22	108.5 (6)
O4—C5—C6	106.3 (7)	O16—C21—H21A	110.0
O4—C5—H5A	110.5	C22—C21—H21A	110.0
C6—C5—H5A	110.5	O16—C21—H21B	110.0
O4—C5—H5AB	110.5	C22—C21—H21B	110.0
C6—C5—H5AB	110.5	H21A—C21—H21B	108.4
H5A—C5—H5AB	108.7	O17—C22—C21	107.9 (7)
O5—C6—C5	108.6 (7)	O17—C22—H22A	110.1
O5—C6—H6A	110.0	C21—C22—H22A	110.1
C5—C6—H6A	110.0	O17—C22—H22B	110.1
O5—C6—H6AB	110.0	C21—C22—H22B	110.1
C5—C6—H6AB	110.0	H22A—C22—H22B	108.4
H6A—C6—H6AB	108.3	O17—C23A—C24A	105.5 (10)
O5—C7—C8	107.2 (8)	O17—C23A—H23A	110.6
O5—C7—H7A	110.3	C24A—C23A—H23A	110.6
C8—C7—H7A	110.3	O17—C23A—H23B	110.6
O5—C7—H7AB	110.3	C24A—C23A—H23B	110.6
C8—C7—H7AB	110.3	H23A—C23A—H23B	108.8
H7A—C7—H7AB	108.5	O17—C23B—C24B	110.7 (11)
O6—C8—C7	109.1 (7)	O17—C23B—H23C	109.5
O6—C8—H8A	109.9	C24B—C23B—H23C	109.5
C7—C8—H8A	109.9	O17—C23B—H23D	109.5
O6—C8—H8AB	109.9	C24B—C23B—H23D	109.5
C7—C8—H8AB	109.9	H23C—C23B—H23D	108.1
H8A—C8—H8AB	108.3	O18A—C25A—C26	112.8 (12)
O6—C9—C10	109.2 (7)	O18A—C25A—H25A	109.0
O6—C9—H9A	109.8	C26—C25A—H25A	109.0
C10—C9—H9A	109.8	O18A—C25A—H25B	109.0
O6—C9—H9AB	109.8	C26—C25A—H25B	109.0
C10—C9—H9AB	109.8	H25A—C25A—H25B	107.8
H9A—C9—H9AB	108.3	O18B—C25B—C26	90.5 (12)
O7—C10—C9	109.3 (6)	O18B—C25B—H25C	113.6
O7—C10—H10A	109.8	C26—C25B—H25C	113.6
C9—C10—H10A	109.8	O18B—C25B—H25D	113.6
O7—C10—H10B	109.8	C26—C25B—H25D	113.6
C9—C10—H10B	109.8	H25C—C25B—H25D	110.8
H10A—C10—H10B	108.3	O13—C26—C25B	106.3 (14)
O7—C11—C12	109.4 (7)	O13—C26—C25A	106.9 (12)
O7—C11—H11A	109.8	O13—C26—H26A	110.3
C12—C11—H11A	109.8	C25A—C26—H26A	110.3
O7—C11—H11B	109.8	O13—C26—H26B	110.3
C12—C11—H11B	109.8	C25A—C26—H26B	110.3
H11A—C11—H11B	108.2	H26A—C26—H26B	108.6
O2—C12—C11	107.1 (6)	O13—C26—H26C	110.5
O2—C12—H12A	110.3	C25B—C26—H26C	110.5
C11—C12—H12A	110.3	O13—C26—H26D	110.5
O2—C12—H12B	110.3	C25B—C26—H26D	110.5
C11—C12—H12B	110.3	H26C—C26—H26D	108.7
H12A—C12—H12B	108.5	F8—C27—F9	109.0 (7)
F3—C13—F2	109.3 (7)	F8—C27—F7	106.4 (8)
F3—C13—F1	107.0 (8)	F9—C27—F7	106.1 (8)
F2—C13—F1	107.3 (9)	F8—C27—S3	113.9 (6)
F3—C13—S1	111.0 (7)	F9—C27—S3	111.6 (6)
F2—C13—S1	110.2 (6)	F7—C27—S3	109.5 (5)
F1—C13—S1	111.8 (6)	F11—C28—F10	109.1 (7)
F4—C14—F5	107.5 (6)	F11—C28—F12	108.8 (6)
F4—C14—F6	107.4 (6)	F10—C28—F12	109.3 (6)
F5—C14—F6	102.9 (6)	F11—C28—S4	111.0 (5)
F4—C14—S2	117.6 (6)	F10—C28—S4	108.7 (5)
F5—C14—S2	109.5 (5)	F12—C28—S4	109.9 (6)
F6—C14—S2	111.0 (4)	H12C—O12—H12D	101 (5)
H1C—O1—H1D	93 (4)	H12C—O12—H12E	103 (5)
H1C—O1—H1E	108 (5)	H12D—O12—H12E	110 (5)
H1D—O1—H1E	97 (4)	C26—O13—C15	109.0 (9)
C1—O2—C12	112.9 (6)	C17—O14—C16	113.0 (8)
C2—O3—C3	111.4 (6)	C18—O15—C19	112.1 (7)
C4—O4—C5	111.2 (7)	C21—O16—C20	111.7 (6)
C7—O5—C6	114.3 (7)	C22—O17—C23A	118.1 (9)
C9—O6—C8	114.0 (7)	C22—O17—C23B	106.2 (8)
C11—O7—C10	110.0 (6)	O18A—C24A—C23A	109.3 (10)
S2—N1—S1	125.7 (4)	O18A—C24A—H24A	109.8
O8—S1—O9	118.5 (3)	C23A—C24A—H24A	109.8
O8—S1—N1	108.3 (4)	O18A—C24A—H24B	109.8
O9—S1—N1	115.5 (3)	C23A—C24A—H24B	109.8
O8—S1—C13	103.9 (4)	H24A—C24A—H24B	108.3
O9—S1—C13	104.9 (4)	C24A—O18A—C25A	126.5 (11)
N1—S1—C13	104.1 (4)	O18B—C24B—C23B	106.5 (11)
O10—S2—O11	118.7 (3)	O18B—C24B—H24C	110.4
O10—S2—N1	108.7 (3)	C23B—C24B—H24C	110.4
O11—S2—N1	116.5 (3)	O18B—C24B—H24D	110.4
O10—S2—C14	102.1 (3)	C23B—C24B—H24D	110.4
O11—S2—C14	106.7 (4)	H24C—C24B—H24D	108.6
N1—S2—C14	101.7 (3)	C24B—O18B—C25B	101.0 (11)
O13—C15—C16	108.4 (10)	S4—N2—S3	125.1 (4)
O13—C15—H15A	110.0	O19—S3—O20	118.7 (3)
C16—C15—H15A	110.0	O19—S3—N2	116.9 (3)
O13—C15—H15B	110.0	O20—S3—N2	108.6 (3)
C16—C15—H15B	110.0	O19—S3—C27	104.5 (4)
H15A—C15—H15B	108.4	O20—S3—C27	103.9 (4)
O14—C16—C15	108.1 (9)	N2—S3—C27	101.8 (3)
O14—C16—H16A	110.1	O21—S4—O22	118.8 (3)
C15—C16—H16A	110.1	O21—S4—N2	117.0 (3)
O14—C16—H16B	110.1	O22—S4—N2	107.8 (3)
C15—C16—H16B	110.1	O21—S4—C28	104.3 (3)
H16A—C16—H16B	108.4	O22—S4—C28	104.0 (3)
O14—C17—C18	107.8 (8)	N2—S4—C28	102.6 (3)

### Hydrogen-bond geometry (Å, º)

**Table:**

*D*—H···*A*	*D*—H	H···*A*	*D*···*A*	*D*—H···*A*
O1—H1*C*···O5	1.00 (3)	1.90 (6)	2.690 (7)	134 (6)
O1—H1*C*···O6	1.00 (3)	2.08 (6)	2.898 (8)	138 (6)
O1—H1*D*···O2	1.01 (3)	2.19 (7)	2.835 (7)	120 (5)
O1—H1*D*···O7	1.01 (3)	1.96 (7)	2.687 (7)	127 (6)
O1—H1*E*···F5^i^	1.03 (3)	2.14 (6)	2.989 (7)	139 (6)
O1—H1*E*···F6^i^	1.03 (3)	2.12 (4)	3.065 (7)	152 (6)
O12—H12*C*···F11^ii^	1.00 (3)	2.50 (4)	3.436 (8)	154 (7)
O12—H12*D*···O13	0.97 (3)	1.85 (6)	2.666 (9)	140 (7)
O12—H12*D*···O18*A*	0.97 (3)	2.36 (6)	3.079 (12)	131 (6)
O12—H12*D*···O18*B*	0.97 (3)	1.97 (6)	2.753 (15)	137 (7)
O12—H12*E*···O14	0.97 (3)	2.14 (7)	2.831 (8)	128 (6)
O12—H12*E*···O15	0.97 (3)	1.95 (6)	2.718 (7)	135 (6)

Symmetry codes: (i) *x*, *y*, *z*−1; (ii) *x*, *y*, *z*+1.

## References

[bb1] Higashi, T. (1995). *ABSCOR*. Rigaku Corporation, Tokyo, Japan.

[bb2] Kitada, A., Takeoka, S., Kintsu, K., Fukami, K., Saimura, M., Nagata, T., Katahira, M. & Murase, K. (2018). *J. Electrochem. Soc.* **165**, H121–H127.

[bb3] Rigaku (2006). *RAPID-AUTO*. Rigaku Corporation, Tokyo, Japan.

[bb4] Sheldrick, G. M. (2015*a*). *Acta Cryst.* A**71**, 3–8.

[bb5] Sheldrick, G. M. (2015*b*). *Acta Cryst.* C**71**, 3–8.

